# Editorial: Combatting tropical diseases: a multi-level approach

**DOI:** 10.3389/fcimb.2024.1406964

**Published:** 2024-04-23

**Authors:** Santanu Sasidharan, Guillermo R. Labadie

**Affiliations:** ^1^ Department of Physics and Astronomy, The University of British Columbia, Vancouver, BC, Canada; ^2^ Instituto de Química Rosario (IQUIR) - Consejo Nacional de Investigaciones Científicas y Técnicas (CONICET), Rosario, Santa Fe, Argentina; ^3^ Facultad de Ciencias Bioquímicas y Farmacéuticas, Universidad Nacional de Rosario, Rosario, Santa Fe, Argentina

**Keywords:** neglected tropical diseases, vaccines, drug discovery, drug development, infectious diseases

COVID-19 has shown us the devastating effects of infectious diseases around the world. To date, the virus has claimed 7 million lives, with new variants emerging every few months (COVID-19 cases, 2023). Concurrently, as the world grapples with the pandemic, the burden of tropical diseases has surged dramatically. It is a sad scenario where tropical diseases, which affect more than 2 billion people and claim nearly 2 million lives every year, are often overlooked. It is only a matter of time before disease-causing microorganisms evolve from being epidemics to pandemics.

Tropical diseases predominantly afflict developing countries with tropical and subtropical climates. The weather is conducive to the growth and transmission of the disease-causing microorganisms, which often remain in animal reservoirs for long periods of time. Recent increases in human-animal contact, poor hygiene, migrations and international trade have increased the risk of disease breakouts and spillovers. The limited number of available drugs for these neglected diseases has not been able to control these pathologies effectively and the frequency of relapses has in turn increased the burden on the healthcare systems. With the exception of the few vaccines awaiting FDA approval, there are no available vaccines for tropical diseases (Vuitika et al., 2022). Another major concern is the increasing number of drug-resistant strains of these microorganisms which cripples the elimination programs. Hence, it is imperative for researchers and governments to collaborate closely to combat, mitigate and eradicate these maladies.

We have designed this Research Topic to highlight the latest advances in the fight against tropical diseases. The Research Topic includes three articles on the prevention of viral diseases: dengue and hepatitis B. The review by Norshidah et al., focuses on the development of antivirals targeting the NS2B/NS3 protease of the dengue virus by examining 105 studies from 2015-2022 in depth. The review discusses the need to integrate *in silico* and *in vitro* screening for better antivirals against dengue virus. The second dengue article by Qin et al., shows the antiviral activity of *Brucea javanica* extracts and demonstrates that the oil emulsion is able to suppress hepatitis B virus replication by effectively upregulating IL-6 production. Interestingly, bruceine B, a major component of *B. javanica* seeds, was identified as the active agent responsible for IL-6 induction and hepatitis B virus inhibition. Bourgeois et al. in their study on dengue virus, evaluated kinase regulation using a machine learning approach that uses drug target information and a small drug screening to predict kinase regulators. They demonstrated that receptor tyrosine kinases are upregulated in dengue-infected cells and that knockouts of the kinases reduce infection in hepatocytes. It is important to understand from these studies that *in silico* screening coupled with *in vitro* assays of natural products can help identify new therapeutic agents against infectious diseases.

In addition to viruses, parasites are also known to cause disease and are responsible for different neglected diseases. One such parasite is *Toxoplasma gondii*, which causes toxoplasmosis. The disease affects both humans and livestock and can even lead to death. Sun et al. developed a recombinant GRA12 protein encapsulated PLGA nanoparticle as a vaccine against acute toxoplasmosis and demonstrated the efficacy of the vaccines in mice. The PLGA-encapsulated GRA12 vaccine was able to increase the IFN-gamma and IL-10 levels while decreasing IL-4 levels in mice, indicating the efficacy of the vaccine. This vaccine candidate holds tremendous potential against acute toxoplasmosis.

In conclusion, this Research Topic summarizes the advances in the development of new drugs and vaccines against infectious diseases. Encompassing viruses and parasites affecting both humans and livestock. The articles underscore the urgency for innovative strategies to address neglected tropical diseases worldwide. As editors, we emphasize the need for new and innovative approaches to tackle tropical diseases neglected in the world. We believe that the Research Topic will help researchers understand that a combined *in silico*-*in vitro* approach is essential for the development of drugs and vaccines. Moreover, the need for governmental intervention in the fight against tropical diseases should be highlighted. We extend our gratitude to the editorial team and authors for their invaluable contributions, which have greatly contributed to the success of this Research Topic.

## Author contributions

SS: Conceptualization, Writing – original draft. GL: Conceptualization, Writing – review & editing.

## References

[B1] COVID-19 cases (2023) datadot. Available online at: https://data.who.int/dashboards/covid19/cases (Accessed March 16, 2024).

[B2] Vuitika L. Prates-Syed W. A. Silva J. D. Q. Crema K. P. Côrtes N. Lira A.. (2022). Vaccines against emerging and neglected infectious diseases: an overview. Vaccines 10, 1385. doi: 10.3390/vaccines10091385 36146463 PMC9503027

